# Recombinant phage displaying ToAP2D peptide with antifungal activity against *Sporothrix globosa*

**DOI:** 10.3389/fphar.2022.1022651

**Published:** 2022-10-07

**Authors:** Tianyi Yan, Lin An, Feng Chen

**Affiliations:** ^1^ Department of Rehabilitation Medicine, China-Japan Union Hospital of Jilin University, Changchun, China; ^2^ Department of Dermatology, China-Japan Union Hospital of Jilin University, Changchun, China

**Keywords:** Sporothrix, immune responses, antimicrobial peptides, ToAP2D peptide, recombinant phage

## Abstract

We designed and synthesized recombinant phage nanofibers displaying ToAP2D peptide and investigated their antifungal effect on *Sporothrix* and the corresponding mechanism. Antimicrobial peptide, ToAP2D, was used as the template. The effect of synthesized recombinant phages on the immune function of CD4^+^ T lymphocytes in mice was tested using an enzyme-linked immunosorbent assay. The therapeutic effect and safety of recombinant phage administration on *Sporothrix*-infected BALB/c mice were evaluated based on survival analysis, histopathological changes, and renal and liver functions. The successfully prepared recombinant phage displaying ToAP2D peptides significantly inhibited *Sporothrix* growth. According to the scanning electron microscopy results, the recombinant phage caused shrinkage and rupture of *Sporothrix globosa*, leading to leakage of the contents. The Hoechst/propidium iodide double staining test indicated that the recombinant phage could induce cell apoptosis of *Sporothrix globosa*. The apoptotic pathway might be due to the accumulation of reactive oxygen species in large quantities in cells, activating caspase dependence; this reduced inflammation, prolonged the survival time, and enhanced levels of IFN-γ and IL-17 in mice. We believe that recombinant phage inhibits *Sporothrix* growth by adjusting the immune response of mice, inducing *Sporothrix* apoptosis and improving animal survival. This study offers a new approach to preparing antimicrobial peptides.

## Introduction

*Sporothrix* can cause local skin infection and even severe systemic disseminated infection. Traditional drug therapy is still the main therapy against sporotrichosis (Barros et al., 2011). Itraconazole (ITR) is a broad spectrum triazole antifungal drug. However, antifungal drugs must be used for extended periods and are prone to the emergence of *Sporothrix globosa*-resistant strains (Donnelly et al., 2008). In addition, they pose potential health risks to patients, especially patients with liver and kidney injury, children, and pregnant women (Tuccori et al., 2008). Therefore, the development of novel antifungal therapies is necessary. Among these, antifungal nanomaterials are promising (Minakshi et al., 2019).

Antimicrobial peptides (AMPs) are small peptides widely distributed in natural organisms. They boast a relatively broad spectrum and selective toxicity and do not easily develop drug resistance. They can selectively inhibit or kill bacteria, fungi, viruses, and other pathogenic microbes (Gao et al., 2018). In addition, they can promote cell healing and immunoregulation, demonstrating the potential for the development of new antifungal drugs. However, AMPs, especially natural AMPs, have relatively large molecular weights, weak antifungal activity, poor stability, hemolytic toxicity, complicated extraction processes, and other disadvantages (Gao et al., 2018). Most small-molecule AMPs from insects have low antifungal activity, are easy to degrade, and cannot maintain effective concentrations for a long time (Jenssen et al., 2006). Some AMPs exhibit strong antigenicity. These limitations limit the applications of AMPs. Transformation of the structure of natural AMPs, including peptide chain length and specific amino acids, can improve pathogenic fungi inhibitory activity while reducing their toxicity so that they may have immunogenicity but no antigenicity (Gao et al., 2018), thus improving their antifungal action.

Phages are bioactive nanomaterial. Exogenous genes can be inserted into the genes of filamentous phages so that target polypeptides can be displayed on the surface of the phage in the form of a fusion protein. Phages are harmless to humans, safe, simple to prepare, and inexpensive, making them suitable for large-scale development and application (Rehman et al., 2019). Different antigen-binding polypeptides can be fully displayed on the surface of phages in the form of multicopy, maintaining the natural conformation and stability of polypeptides, thus better inducing high antifungal biofilm activity. Therefore, antigen-binding polypeptides demonstrate significant potential for the development of new antibiotics (Chen et al., 2017; Chen et al., 2019). *Sporothrix* has biological properties that are closest to those of *Candida albicans*. Therefore, we used its structure as a template and transformed it into three segments of small-molecule AMPs, ToAP2A (GSKLIPGVMKLFSKK), ToAP2C (GAKLIPGVMKLFRKK), and ToAP2D (GDKLIPGVMKLFRKK, GK), using a reasonable molecular design. The killing effects of the three scorpion venom-derived AMPs after molecular genetic improvement on *Sporothrix* cultured in an isolated manner were then compared using *in vitro* experiments. The results showed that ToAP2D showed the highest anti-*Sporothrix* activity, good serum stability, and no acute toxicity, indicating its potential to be developed as a new anti-*Sporothrix* drug (Yan et al., 2021).

We first inserted gene fragments of the improved AMP ToAP2D into phage genes, prepared many AMPs using phage display techniques, and then passively inoculated the same as *Sporothrix*-infected mice. We also evaluated its antifungal, organ-inflammation-reducing, and animal survival-improving effects.

## Materials and methods

### Animals

All experimental protocols were approved by the Ethics Committee of the Institute for Laboratory Animal Research of the China-Japan Union Hospital of Jilin University (Jilin, Changchun) and were implemented in accordance with the guidelines for laboratory animal management of Jilin University. Female BALB/c mice (8–10-week-old; body weight: 16–20 g) were purchased from Yisi Laboratory Animal Technology Co., Ltd. (Changchun, Jilin, China). To reduce pain in the experimental animals, all operations were performed under adequate anesthesia with phenobarbital.

### Strains and culture conditions

*Sporothrix* used in the experiment was isolated from a patient with disseminated sporotrichosis. The separated strains underwent strict transformation culture and molecular characterization and were identified as *Sporothrix globosa*. *Sporothrix globosa* was first cultured on Sabouraud’s medium at 25°C. Seven days later, the cultured fungi were transferred to brain heart infusion to culture at 37°C for 7 days with a shaker speed of 150 rpm. The culture fluid was centrifuged, resuspended in PBS, and diluted to 1 × 10^8^ cells/mL.

### Antimicrobial peptide synthesis

We previously reported ToAP2D, a 15-amino acid peptide (Antifungal Activity of ToAP2D 2021), which showed higher antifungal activity against *S. globosa*. Antifungal peptide ToAP2D was synthesized by ChinaPeptides Co., Ltd. (Suzhou, China), and its purity was determined to be higher than 95% using reversed-phase high-performance liquid chromatography and electrospray ionization mass spectrometry.

### Construction of recombinant phage

Fuse-55, the phage vector, was reproduced in the *E. coli* TG1 strains. The wild phages used in this experiment were maintained in our laboratory. Construction of the recombinant phage vector fuse-55 and recombinant phage are described in the corresponding literature (Malik et al., 1996; Yang et al., 2005). Briefly, the synthesized complementary oligonucleotides encoding peptide GK were connected to BglI-digested fuse-55. *E. coli* TG1 transfected with the recombinant phage vector inserted into the peptide was cultured in Luria-Bertani liquid medium containing ampicillin. The culture supernatant containing phage particles was collected and precipitated twice in polyethyleneglycol-6000 (PEG-6000; final concentration: 5%; 0.5M NaCl) for 12 h. The phage particles were then resuspended in 2 ml of phosphate-buffered saline (PBS). The concentration of phage particles was determined using a spectrophotometer (OD at 270 nm was 1.0, corresponding to 0.26 μg/μL).

### Western blotting

Antibodies to AMPs displayed on bacteriophages were purchased from China Peptides Co., Ltd. (Suzhou, China). The protein was extracted from the recombinant phage, denatured, electrophoresed, and transferred onto the PVDG membrane, which was then placed in Tris-tricine buffer saline (TBST). Subsequently, the protein membrane was blocked with 3% skim milk powder at 37°C for 60 min, followed by membrane washing with TBST four times for 3 min each. Next, the nitrocellulose membrane was incubated with 1:2000 diluted serum in TBST and 5% skim milk at 37°C for 1 h. After adding oxidase-coupled goat anti-mouse IgG (Vector Laboratories Inc., United States), the mixture was incubated at 37°C for 1 h. Finally, the membrane was put into the chemiluminescence-based image analysis system for development for 3 min.

### *In Vitro* bacteriostasis experiment

A total of 50 μL of bacterial solution (concentration: 1 × 10^8^ cells/mL) was coated onto broth solid medium, and autoclaved filter paper (0.5 cm in diameter) was pasted onto the surface of the solid medium. Then, 6 μL of hybrid phage expressing peptide GDKLIPGVMKLFRKK (phage-GK (PG)) (concentration: 4, 2, and 1 mg/ml by AMP concentration) and the control sample were dropped onto the filter paper and left in an incubator at 37°C for inverted culture for 5–7 days; the zone of inhibition was then observed.

### Scanning electron microscopy (SEM)

The strains were divided into experimental and control groups; the former used a laser, while the latter was not illuminated. The two groups were cultured for 4 h in a 24-well plate on a cell culture slide. The supernatant was discarded, and each well was rinsed in PBS once and then fixed with 2.5% glutaraldehyde (0.2 M PBS, pH 6.0) for 1 h at 4°C. After fixation, the disks were rinsed three times with PBS and dehydrated in a graded series of ethanol (30, 50, 70, 80, and 90% for 7 min each and 100% for 10 min). The disks were critical point dried prior to being coated with a gold sputter and observed using SEM.

### Cell apoptosis study

The strains were centrifuged at 5,000 × g for 5 min. Next, the cells were collected and resuspended in 800 μL of cell staining buffer. Hoechst 33342 (5 µL) staining solution was added to the solution and mixed well by turning it upside down. Then, 5 μL of propidium iodide (PI) was added to the solution and mixed as described above. The solution was then placed in an ice bath for 30 min. It was then rinsed once with PBS, smeared, and observed under a laser scanning confocal microscope (LSCM).

### Measurement of Mitochondrial Membrane Potential (MMP)

*Sporothrix globosa* treated with the recombinant phage (1 × 10^8^ CFU/ml) was washed and resuspended in PBS (pH 7.4). Then, JC-1 was added to the mixture at a final concentration of 5 μM, followed by incubation at 37°C for 20 min. Fluorescence absorption was detected using a fluorescence spectrophotometer at an excitation and emission wavelength of 490 and 530 nm, respectively.

### Measurement of oxidative stress

DCFH-DA was diluted in yeast extract-peptone-dextrose (YPD) liquid medium at 1:1,000 to a final concentration of 10 μM. The *Sporothrix* solution was obtained after overnight culture, and the cells were collected and suspended in diluted DCFH-DA. The cells, at a concentration of 1 × 10^8^ cells/mL, were left for incubation at 37 °C for 20 min. The cells were washed with YPD liquid medium three times. Cells in the experimental and control groups were stimulated directly with reactive oxygen species (ROS)-positive controls and solvents. After 20 min, fluorescence absorption was measured using a fluorescence tester at an excitation and emission wavelength of 490 and 530 nm, respectively. ROS accumulation was calculated using the formula: (F_test_-F_blank_)/(F_control_-F_blank_), where F_test_ is the sample fluorescence value after laser treatment, F_control_ is the sample fluorescence value in the PBS treatment group, and F_blank_ is the fluorescence value in wells without cells.

### Detection of metacaspase activation

After laser treatment, the *Sporothrix* solution from the experiment and control groups was centrifuged at 2000 × g for 3 min, and the cells were resuspended. Then, the cells were stained with 10 μM of CaspACE FITC-VAD-FMK for 20 min at 37°C, followed by rinsing with PBS twice. The cells were then smeared and observed under LSCM. The fluorescence absorption was tested using a fluorescence tester at an excitation and emission wavelength of 494 and 530 nm, respectively.

### Treating *Sporothrix*-Infected Mice with Recombinant Phage

The mouse model of disseminated *Sporothrix globosa* was administered an intravenous injection of 0.2 ml (1 × 10^8^ cells/mL) of *Sporothrix globosa* suspension. Mice were randomly divided into four groups, each containing six mice. One day after infection, the following drugs were injected into the tail vein of each mouse every 3 days (6 injections in total): (1) 25 μg of PG in 100 μL PBS (PG group), (2) 25 μg of wild-type (WT) phage particles in 100 μL PBS (mock group), (3) ITR (5 mg/kg body weight, ITR group), or (4) PBS alone (PBS group; negative control). The mice were sacrificed on days 8 and 20 of infection.

### Enzyme-linked immunosorbent assay (ELISA)

On days 8 and 20, the mouse sera were isolated and frozen at −80°C before use. Mouse serum IFN-γ and IL-17 levels were measured using Mlbio (Shanghai, China) ELISA kits, according to the manufacturer’s instructions. Absorbance was measured at 450 nm using a 96-well plate reader.

### Lung histopathology

Mouse lung tissues were collected from each group and fixed in 10% buffered formalin for 24 h. After dehydrating and embedding in paraffin, the tissues were sectioned at approximately 5 µm using a paraffin slicer. Subsequently, the sections were stained with H&E to analyze the degree of inflammatory cell infiltration in the lung tissues, and the ImageJ software was used to quantify the percentage area of inflammatory cells on day 20. Neutrophils have very characteristic nuclei with three to five lobes connected by thin strands; and the size of neutrophils is about 10–15 µm. Lymphocytes are small cells, 8 to 10 microns in diameter and have large round nuclei.

### Survival analysis

Mice were randomly divided into four groups of 10 mice each. *Sporothrix globosa* suspension (0.2 ml; 5 × 10^8^ cells/mL) was injected into the tail vein of mice. On day 1 after infection, the mice were intraperitoneally injected with the above drugs. We observed a survival time of 2 weeks after treatment.

### Evaluation of liver and renal function

The mice were randomly divided into two groups of seven mice each. The experimental group was injected with 25 μg of PG in 100 μL of PBS into the tail vein, and the control group was injected with 100 μL of PBS. Blood was drawn from the tail vein on day 3, and aspartate aminotransferase (AST), alanine aminotransferase (ALT), blood urea nitrogen (BUN), and creatinine (Cr) levels were measured.

### Statistical analysis

Statistical analyses were performed using SPSS v24 (SPSS, Chicago, IL, United States). Values are expressed as the mean ± standard error of the mean for normally distributed data. One-way analysis of variance (ANOVA) and the Kruskal–Wallis H or Mann–Whitney U tests were performed to evaluate between-group differences. We examined the differences in survival time between different groups using the Kaplan-Meier test. The criterion for statistical significance was set at *p* < 0.05.

## Results

### Preparation of recombinant phage

**FIGURE 1:**
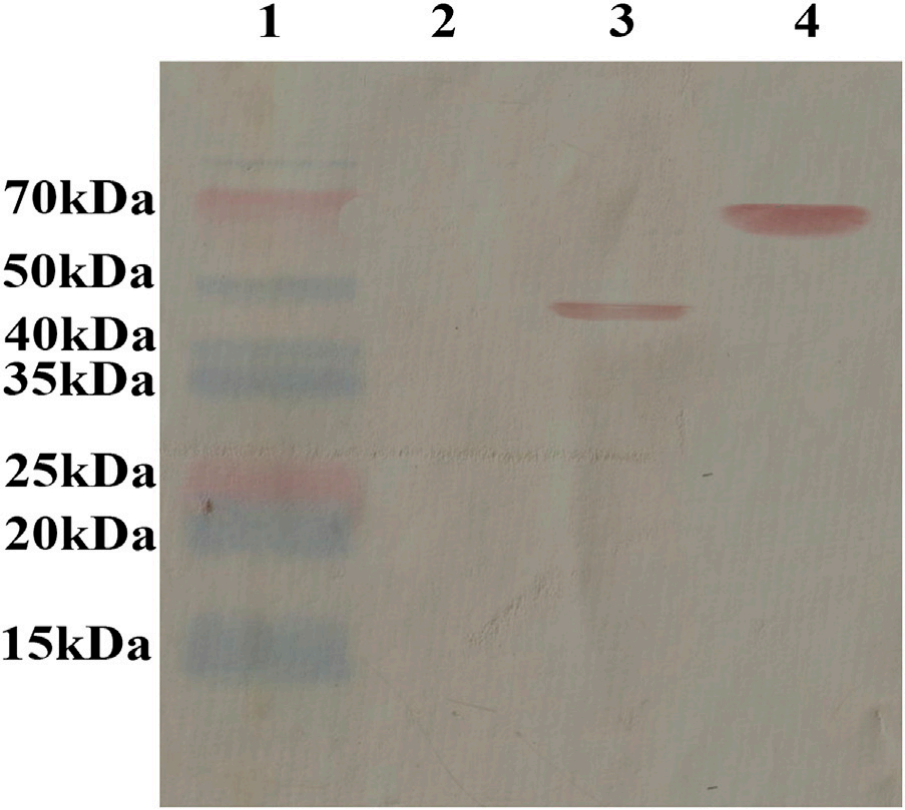
Protein expression of PG detected using western blotting. The antimicrobial peptide antibody reacted with the fusion protein in the hybrid phage, and the results showed that the antimicrobial peptide GK protein was displayed on the surface of the recombinant phage. Lane 1: marker (P12083); Lane 2: WT phage; Lane 3: recombinant phage displaying the GDKLIPGVMKLFRKK peptide; Lane 4: BSA link to GDKLIPGVMKLFRKK peptide.

The expression of PG was tested using western blotting. According to the reaction of AMP antibodies with fusion proteins in the hybrid phage, AMP-GK was displayed on the surface of the recombinant phage (Figure 1).

### *In Vitro* antifungal experiment

The inhibitory effect of PG on *Sporothrix globosa* was analyzed using the bacteriostatic circle test. The results showed that the recombinant phage had no obvious antifungal effects at a concentration of 1 mg/ml. When the concentration was increased to 2 and 4 mg/ml, the recombinant phage had an inhibitory effect on *Sporothrix globosa*. The inhibitory effect was obvious (11.15 ± 1.38 mm) when the concentration of recombinant phage was 4 mg/ml. This suggests that PG (antifungal peptide at a concentration of 4 mg/ml) has an effective antifungal activity against *S. globosa* (Table 1). Therefore, we used PG (antifungal peptide at a concentration of 4 mg/ml) for our experiments.

**TABLE 1:** Antifungal diameter of antimicrobial peptides *in vitro*.

	Concentration (mg/ml)		
	4	2	1
**ToAP2D**	11.15 ± 1.38***	8.96 ± 0.85***	5.00 ± 0.00
**Control**	5.00 ± 0.00	5.00 ± 0.00	5.00 ± 0.00

Normally distributed data are shown as the mean ± SD.***mean compared to control sample, *p* < 0.001.

### Morphological changes in *S. globosa* after recombinant phage treatment in vitro

**FIGURE 2:**
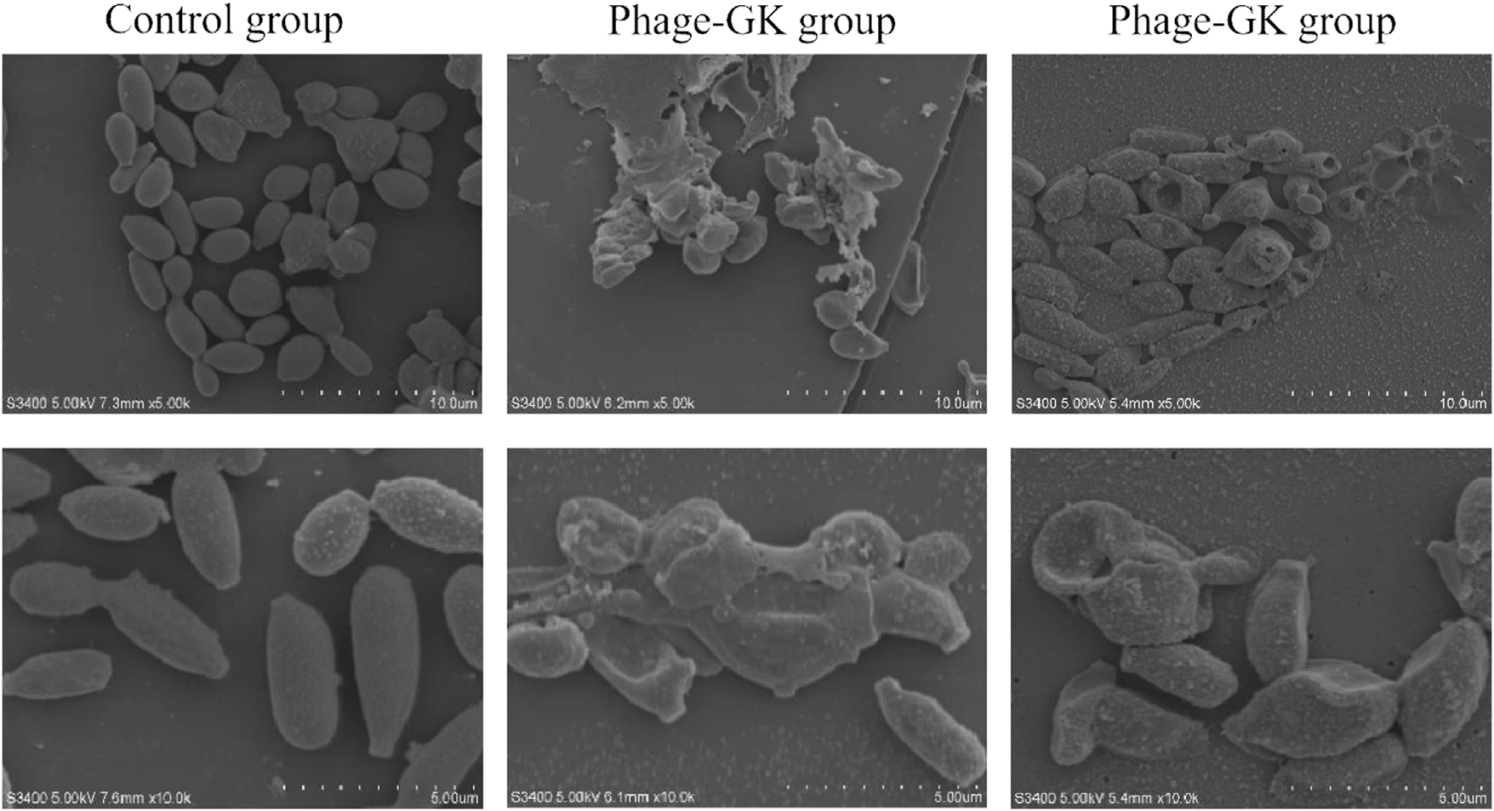
Control group under a scanning electron microscope. The normal morphology of *S. globosa* cells is oval-shaped and plump. Phage-GK group after PG treatment; *S. globosa* cells are damaged and shriveled.

Morphological changes on the surface of *Sporothrix* cells after recombinant phage treatment were observed using SEM. After the control treatment, *S. globosa* cells showed normal morphology, smooth and complete surface, regular shape, and clear boundaries. However, after recombinant phage treatment, *Sporothrix* became irregular in shape, with shrinkage and rupture of the cell membrane, cross-linked strains, and leakage of cell contents (Figure 2).

### *Sporothrix* cell apoptosis after recombinant phage treatment

**FIGURE 3:**
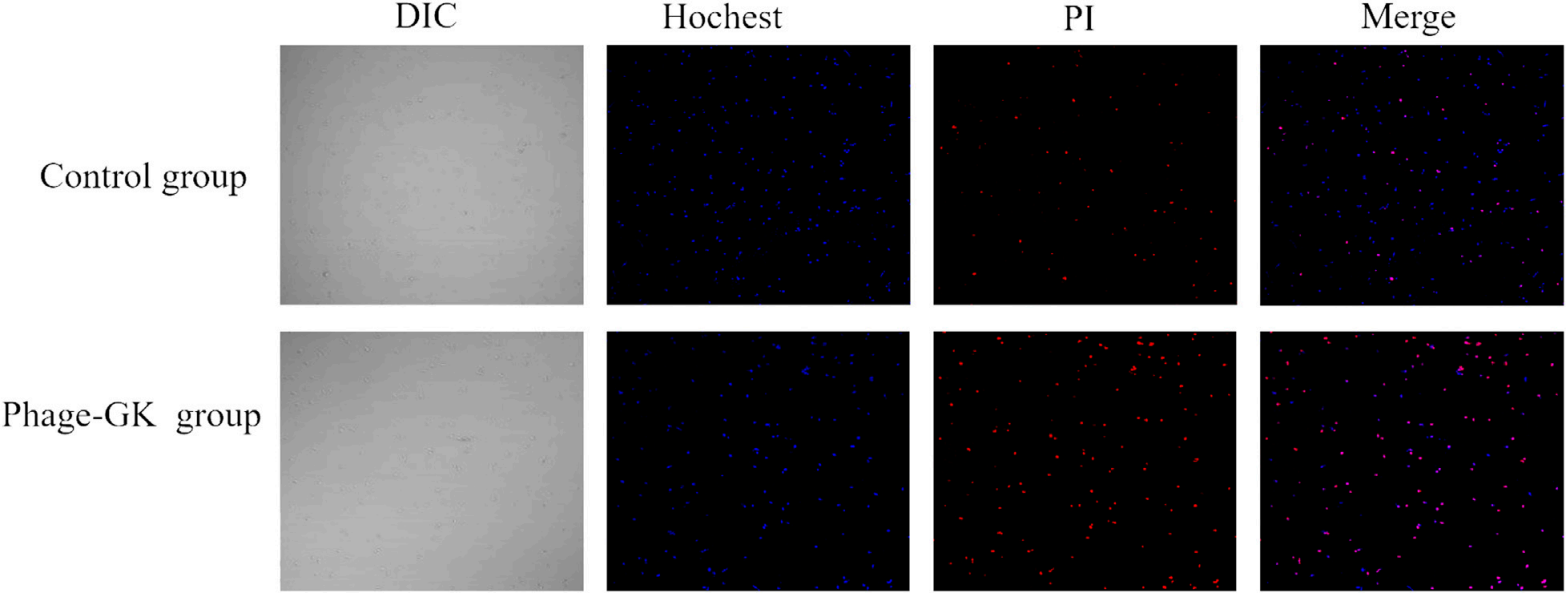
Necrosis and apoptosis of *S. globosa* cells after PG treatment observed using confocal laser scanning microscopy after Hoechst/PI staining.

Hoechst 33342 can penetrate the cell membrane and bind to the chromosomes in apoptotic cells. Apoptotic cells showed significantly enhanced fluorescence compared to normal cells after staining (Yin et al., 2014). However, PI cannot penetrate the cell membrane. Normal or apoptotic cells with complete membranes were not stained. After double staining, under a fluorescence microscope, apoptotic cells showed faint red and strong blue fluorescence; necrotic cells showed strong red and strong blue fluorescence. In the recombinant phage treatment group, strong blue and red fluorescence (Hoechst+/PI+) were observed, indicating both apoptosis and necrosis in *S. globosa*. In contrast, in the control group, only faint blue and red fluorescence (Hoechst+/PI-) were observed, indicating a normal state of *S. globosa* (Figure 3).

### Effect of recombinant phage treatment on MMP in *sporothrix* cells

**FIGURE 4:**
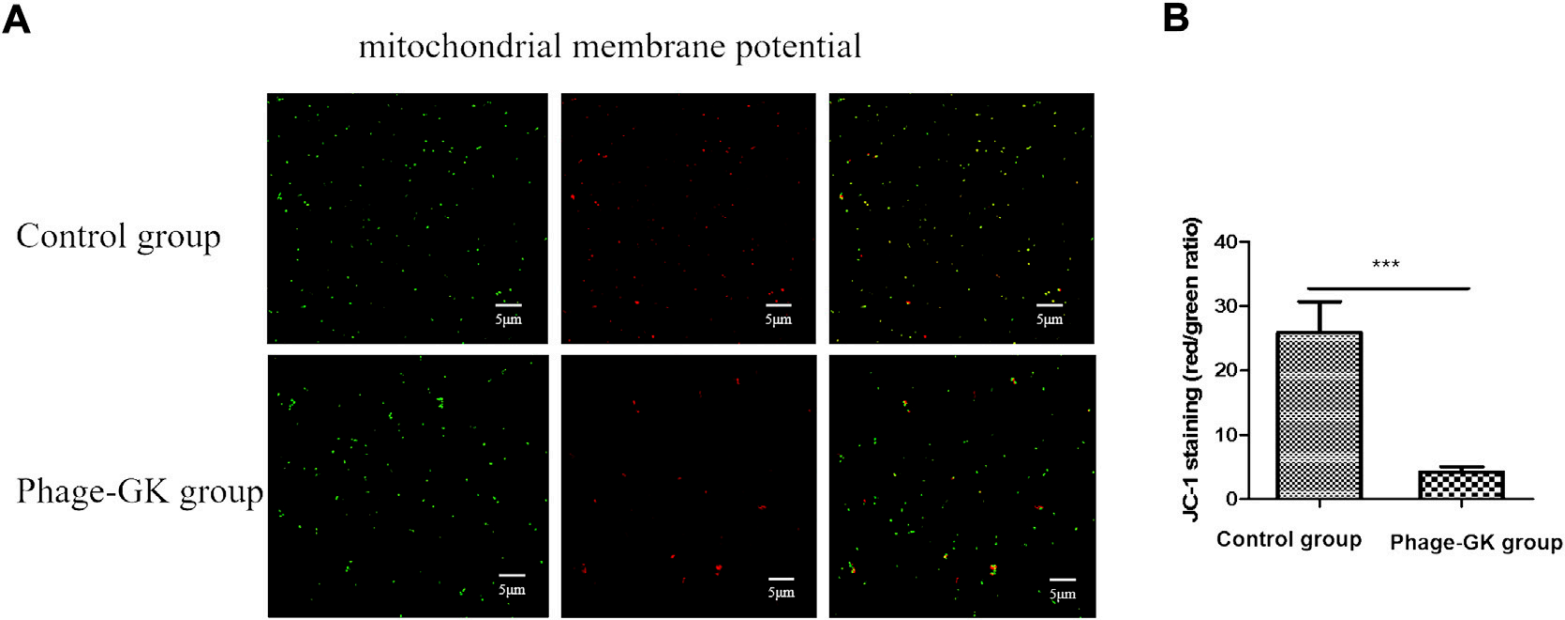
Effects of PG treatment on MMP. **(A)** Confocal laser scanning microscopy to evaluate the effects of PG treatment on MMP in *S. globosa* cells of the study and control groups. **(B)** Flow cytometry was used to detect the MMP. ***p < 0.001 indicates statistical significance.

Mitochondrial dysfunction initiates cell apoptosis (Eisenberg et al., 2007). At a higher mitochondrial membrane potential (MMP), JC-1 accumulates in the mitochondrial matrix and forms J-aggregates, producing red fluorescence. At lower MMPs, JC-1 cannot gather in the mitochondrial matrix but is a monomer that produces green fluorescence. The relative proportions of red and green fluorescence were used to measure mitochondrial depolarization. Through the transition of JC-1 from red to green fluorescence, it is easy to detect a decline in the cell membrane potential. Such a JC-1 transition can serve as an index for testing early cell apoptosis. According to the immunofluorescence results, compared with the control group, the recombinant phage treatment group showed a transition from red to green fluorescence. The fluorescence ratio of *S. globosa* cells in the control group was almost 25.84 ± 1.34, confirming no obvious depolarization. However, the fluorescence ratio of *S. globosa* cells in the recombinant phage group was reduced to 4.24 ± 0.26 (mean ± SEM) (*p* < 0.001), indicating no significant depolarization in *S. globosa* after the treatment (Figures 4A, B). In short, recombinant phages cause a decline in the MMP in *S. globosa*, indicating cell apoptosis.

### Effect of recombinant phage treatment on ROS accumulation in *sporothrix* cells

**FIGURE 5:**
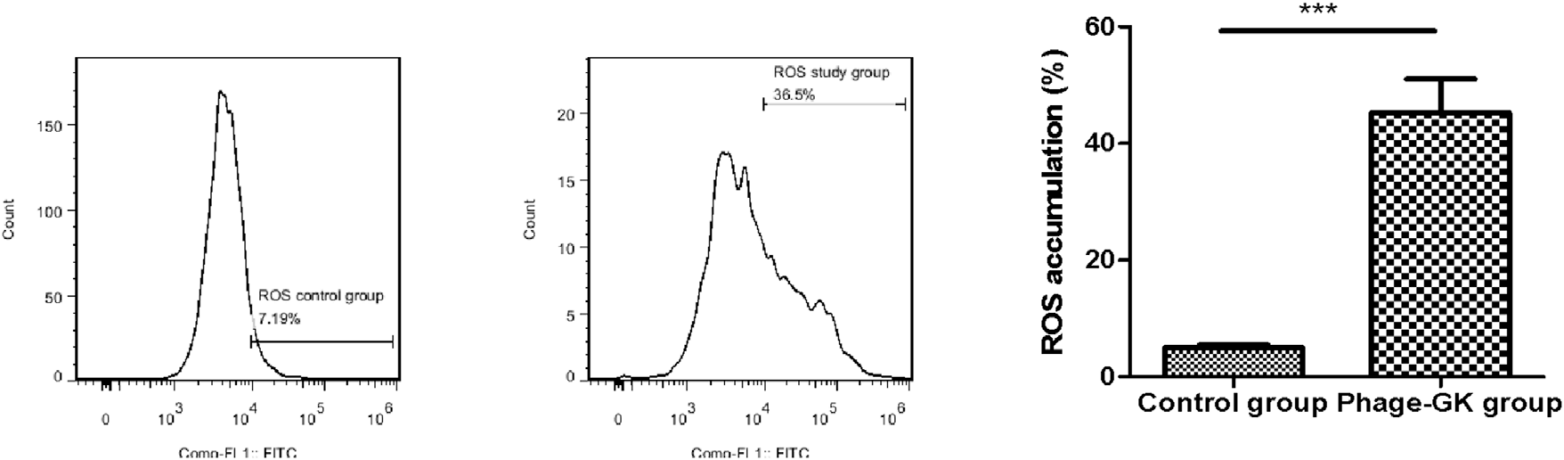
Effect of PG treatment on ROS accumulation in *S. globosa* cells. Flow cytometry was used to detect ROS accumulation. ***p < 0.001 indicates statistical significance.

Mitochondrial depolarization, accompanied by an endogenous increase in ROS, is a characteristic of cell apoptosis (Nury et al., 2021). After recombinant phage treatment, ROS accumulation in cells was significantly improved, without a significant difference from the positive control. No change was observed in ROS accumulation in the control group. This indicates that recombinant phage-induced cell apoptosis may be achieved through the ROS-mediated apoptosis pathway (Figure 5).

### Effect of recombinant phage treatment on caspase activity in *sporothrix*

**FIGURE 6:**
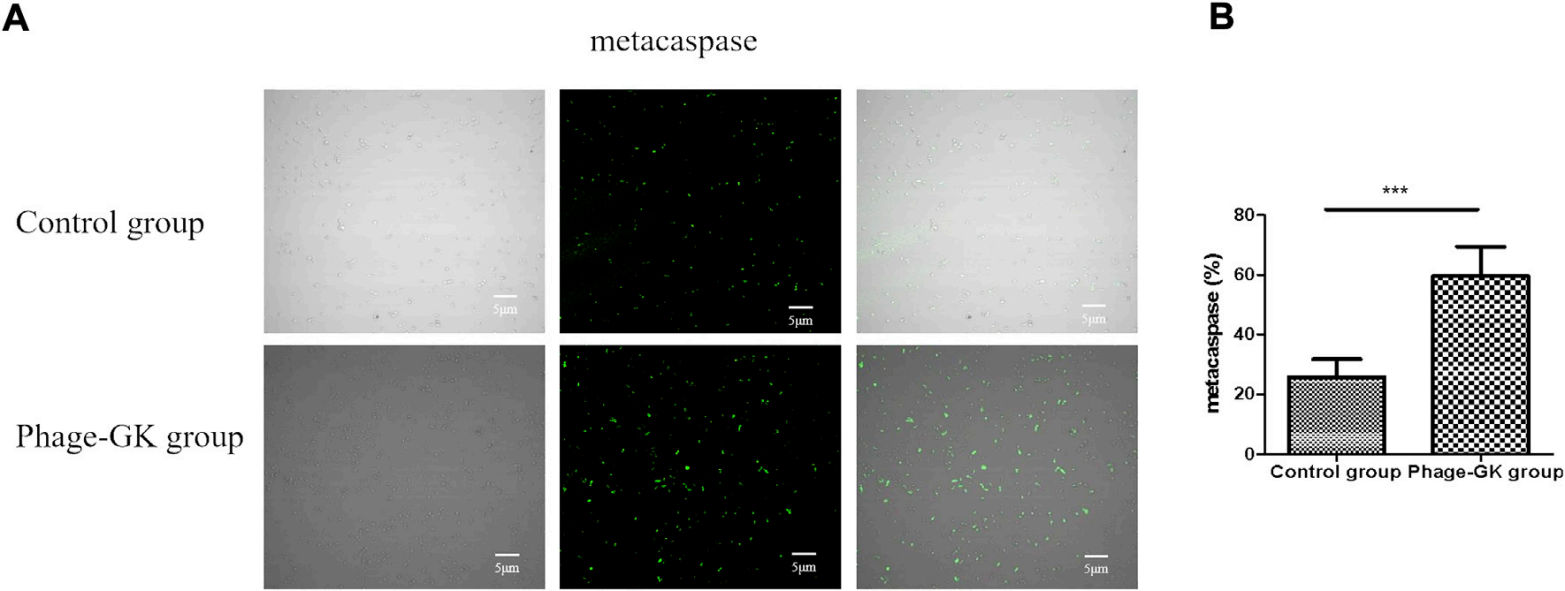
Effects of PG treatment on metacaspase activation. **(A)** Confocal laser scanning microscopy to evaluate the effects of PG treatment on metacaspase activation in *S. globosa* cells of the study and control groups as observed by staining with CaspACE FITC-VAD-FMK. DIC, differential inference contrast. **(B)** Flow cytometry was used to detect the metacaspase activation. ***p < 0.001 indicate statistical significance.

The fungal apoptotic pathway consists of various regulatory factors and effectors (Sharon et al., 2009). In this study, we focused on the caspase apoptotic pathway. Based on the experimental results, a small amount of fluorescence was detected in the blank control. However, in the recombinant phage treatment group, most cells showed bright green fluorescence, indicating that the recombinant phage activated the caspase-dependent cell apoptosis pathway in *Sporothrix* cells (Figure 6).

### Changes in IFN-γ and IL-17 Levels after Phage-GK (PG) Treatment in Mice

**FIGURE 7:**
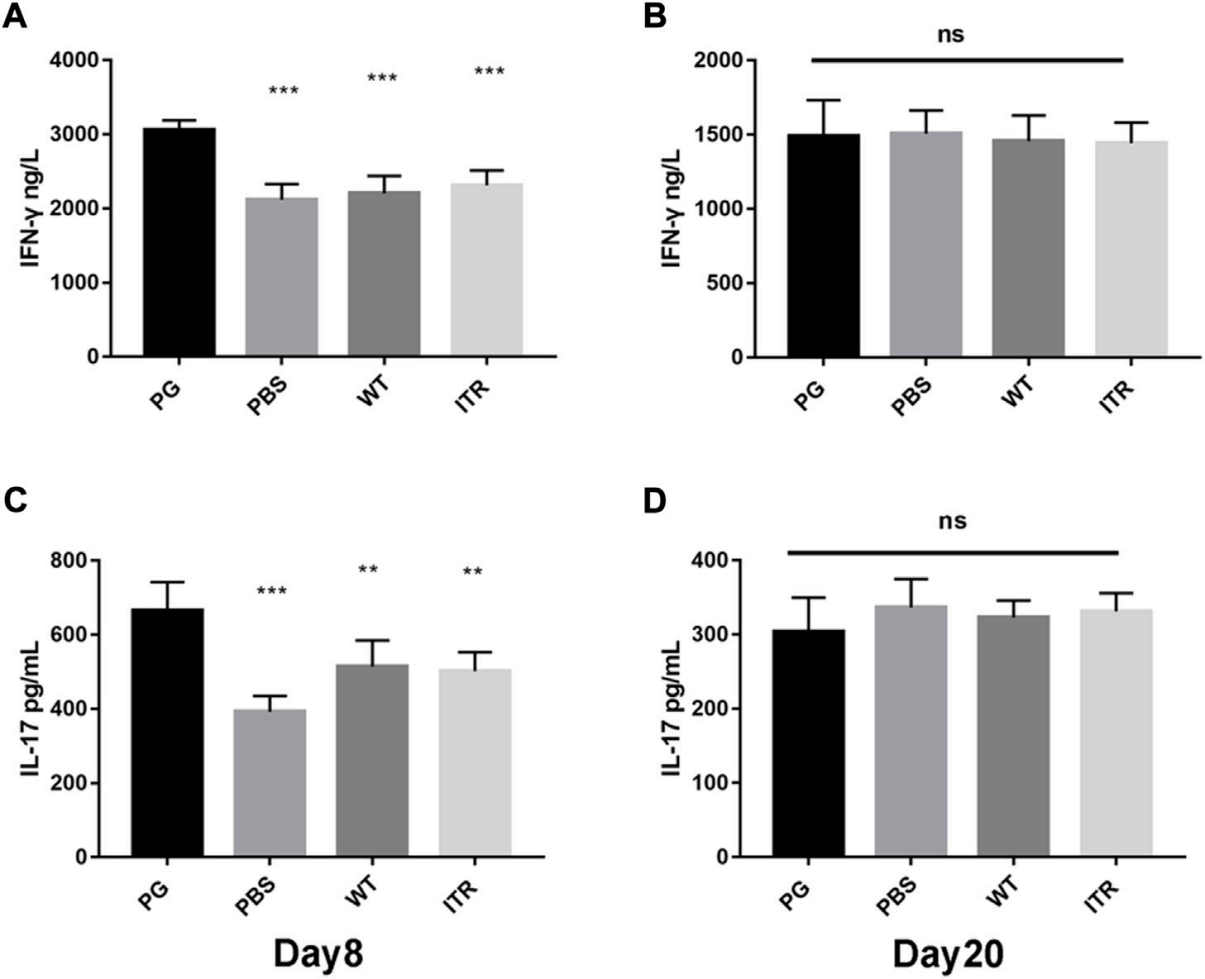
IFN-γ and IL-17 levels after PG treatment in mice. **(A,B)** Change in IFN-γ levels in mice on days 8 and 20 after *S. globosa* infection. **(C,D)** Change in IL-17 levels in mice on days 8 and 20 after *S. globosa* infection. Compared to the levels in mice from the PG group, ***p < 0.001, **p < 0.01, ns: no significance. Results shown are representative of three independent experiments. WT: wild-type phage injection; ITR: itraconazole injection; PG: phage-GK injection.

To assess the development of cell-mediated immunity, the levels of cytokines IFN-γ and IL-17 were assessed using ELISA on days 8 and 20. As shown in Figure 7A and C, the serum IFN-γ and IL-17 levels were significantly higher in the PG group (*p* < 0.001) than in the PBS group on day 8. Serum IL-17 levels were also significantly higher in the PG group than those in the WT and ITR groups (*p* < 0.01). On day 20 (Figures 7B, D), no significant changes in IFN-γ and IL-17 levels were observed in any of the study groups. These results showed that recombinant phage displaying ToAP2D peptide induced IFN-γ and IL-17 expression. IFN-γ and IL-17 are the main cytokines secreted by Th1 and Th17 cells, respectively. We speculated that phage displaying ToAP2D peptide activate Th1 and Th17 responses, promoting antifungal immunity (Figure 7).

### Recombinant phage effectively mitigates inflammation in mice organs

**FIGURE 8:**
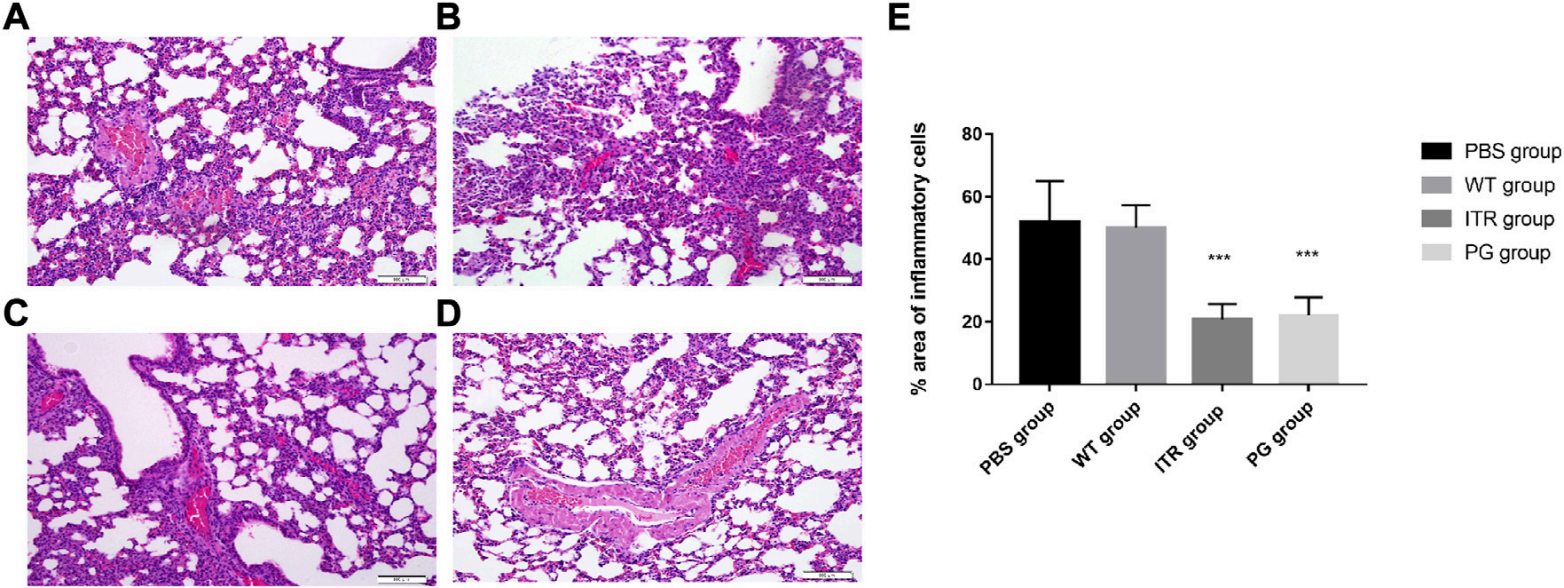
Images of H&E-stained lungs of *S. globosa*-infected mice treated with various formulations on day 20; magnification ×200. **(A)** PBS injection, **(B)** WT injection, **(C)** itraconazole injection, and **(D)** PG injection. **(A,B)** A higher number of inflammatory cells was observed. In contrast, **(C,D)** showed a dramatic decrease in neutrophil and lymphocyte infiltration. **(E)** Compared with the PBS group, the percentage area of inflammatory cells in the different treatment groups on day 20. Results are representative of three independent experiments. WT: wild-type phage injection; ITR: itraconazole injection; PG: phage-GK injection.

The organs showed no obvious lesions as observed under naked eye. However, after H&E staining, on day 8, mice in the PBS (55.37 ± 13.20%) (Figures 8A, E) and wild phage groups (50.34 ± 7.29%) (Figures 8B, E) showed invasion of a large number of lymphocytes and neutrophils in the lungs, indicating significant inflammation. In contrast, in the recombinant phage (24.28 ± 5.69%) (Figures 8D, E) and ITR groups (19.95 ± 4.93%) (Figures 8C, E), lymphocytes and neutrophils of only medium levels were observed, indicating mild inflammation.

### Recombinant phage effectively prolong the survival time of mice with sporotrichosis

**FIGURE 9:**
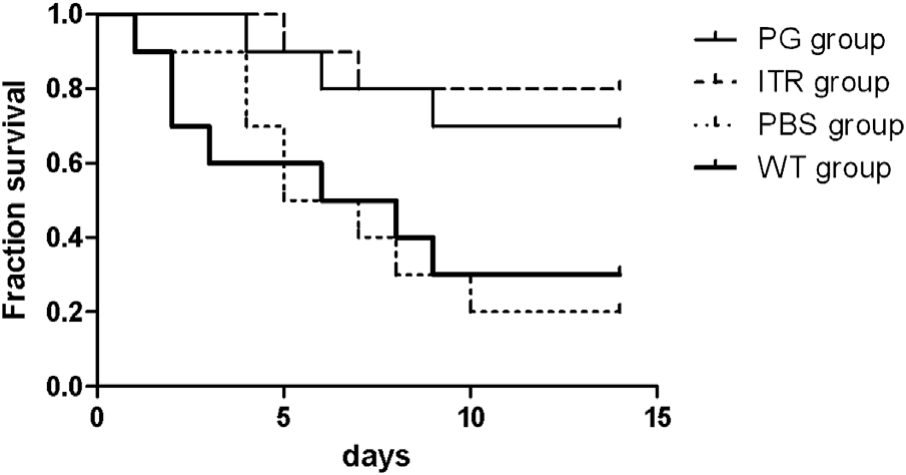
Survival rate of *S.* globosa-infected mice treated with PG, ITR, WT, or PBS alone. WT: wild-type phage injection; ITR: itraconazole injection; PG: phage-GK injection.

The detection time for mouse survival was 14 days (Figure 9). The experimental results showed that mice in the experimental group injected with recombinant phage and ITR showed the highest 14-day survival, reaching 80 and 70%, respectively, with no significant difference. Mice injected with PBS showed significantly reduced 14-day survival (only 20%), and survival of the WT group was 30%. Therefore, the survival rate of the recombinant phage-immunized mice was significantly higher than that of the PBS and WT groups.

### Low toxicity of recombinant phage

**FIGURE 10:**
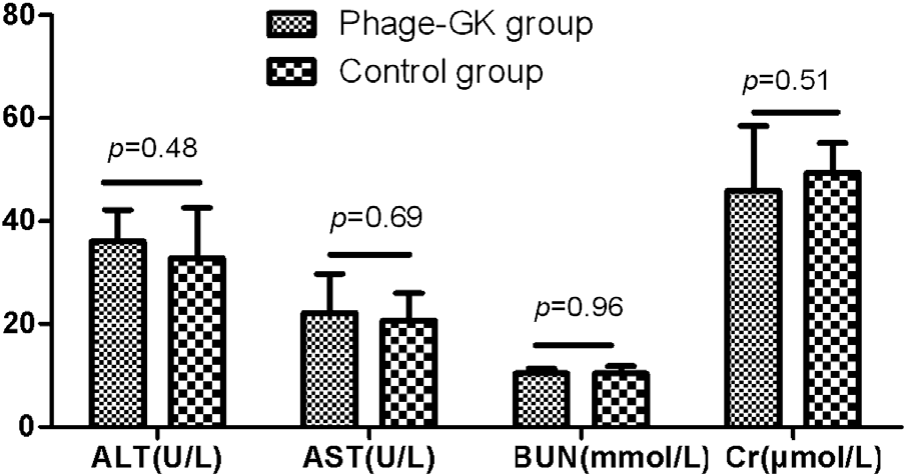
AST, ALT, BUN, and Cr levels after recombinant phage injection. AST, aspartate aminotransferase; ALT, alanine aminotransferase; BUN, blood urea nitrogen; Cr, creatinine.

Mice injected with recombinant phage showed clinical biochemical indexes, including AST, ALT, urea, and creatinine, within normal limits, with no significant difference from the control group (Figure 10).

## Discussion

Sporotrichosis is distributed worldwide, with the number of relevant cases increasing significantly in humans and animals over the past 20 years (Chakrabarti et al., 2015; Queiroz-Telles et al., 2019). Antibiotic resistance has improved worldwide. In contrast, the development of new antibiotics has slowed significantly, increasing interest in new antimicrobial therapy (Barros et al., 2011; García Carnero et al., 2018). The treatment of fungal infections with AMP has received increasing attention. However, due to its large molecular weight, insufficient antifungal activity, poor stability, and other shortcomings, AMP treatment has been limited (Fry, 2018; Gao et al., 2018; Browne et al., 2020). In this study, we improved natural AMPs and displayed improved AMPs on the surface of the phage. This involves a simple preparation process and low cost and yields stable AMPs after restructuring. Based on the results of *in vitro* experiments, recombinant AMPs effectively promoted cell apoptosis and inhibited *Sporothrix* growth. Moreover, recombinant phages had a good therapeutic effect on *Sporothrix*-infected mice.

We explored the effect of recombinant phage treatment on *S. globosa* using LSCM, flow cytometry, SEM, and other experimental methods. After recombinant phage treatment of *S. globosa*, significant shrinkage was observed on the cell surface, with the leakage of cell contents in large amounts, indicating necrosis. Apoptosis and necrosis of *S. globosa* cells were confirmed using Hoechst/PI double staining. Apoptosis has many typical characteristics, abnormal MMP being one of the earliest events. A decline in MMP indicates a change in the mitochondrial membrane structure. According to this research, after recombinant phage treatment, MMP in *S. globosa* declined significantly, thus damaging the mitochondria. Mitochondria are the center for the control of cellular life activities and the regulation of cell apoptosis. Damaged mitochondria lead to abnormal leakage of electrons into the mitochondrial respiratory chain, thereby causing ROS accumulation in the mitochondria. ROS are the main mediating factor of fungal apoptosis, leading to the death of *Sporothrix* by oxidizing biomacromolecules in cells. According to the experimental results, after recombinant phage treatment, *S. globosa* cells showed a significantly enhanced ROS level. This agrees with the previous viewpoint that antifungal agents attack mitochondria by producing excessive ROS, thus inhibiting mitochondrial respiratory function and killing fungi (Kwun & Lee, 2020).

Fungal apoptosis is regulated by various regulators and effector substances (Hamann et al., 2008; Sharon et al., 2009). Currently, there are two apoptotic pathways in yeast: caspase-dependent and caspase-independent. Approximately 40% of fungal apoptosis occurs *via* the first pathway (Sharon et al., 2009). According to relevant research, ROS can activate metacaspases in fungi, leading to fungal apoptosis (Perrone et al., 2008; Tudzynski et al., 2012). Therefore, this experiment further explored the changes in metacaspase activity in *S. globosa* after recombinant phage treatment. According to the results, metacaspase in *S. globosa* was activated after the treatment. Under normal conditions, metacaspases are inactive; however, when external stimuli are present, metacaspases can be activated, causing fungal apoptosis. Therefore, the recombinant phage induced apoptosis in *Sporothrix via* the metacaspase-dependent pathway.

For the first time, we employed PG in sporotrichosis mice and reported that it could increase IFN-γ and IL-17 levels, reducing the severity of infection. T helper (Th) cells reportedly induce antigen-specific T-cell responses in the early stage of inflammation (Cosmi et al., 2014). Th1 and Th17 cell responses are involved in anti-*Sporothrix* infection in the body (Yan et al., 2020). Th1 cell subset is mainly involved in the activation of cellular immunity by secreting IL-2 and IFN-γ, which play important roles in inflammation. IFN-γ inhibits *C. albicans* growth by stimulating the generation of derived cytokines in the body, improving the phagocytic capacity of neutrophils against *C. albicans*, thus enhancing the body’s resistance to *C. albicans* infection (Tarumoto et al., 2014). Th17 cells are also a subset of T lymphocytes that mainly secrete IL-17; they have received increasing attention for their resistance to pathogen infection (Cypowyj et al., 2012; Mear et al., 2014). According to the experimental results, after injection of the recombinant phage, IFN-γ and IL-17 levels in the blood of *Sporothrix*-infected mice increased significantly. Therefore, phage-displaying AMPs can trigger the cellular immune response of Th1 and Th17 cells in the body, thereby achieving fungal resistance. Improvement in the levels of Th1 and Th17 in the blood of infected mice is therefore favorable for the removal of *Sporothrix* and alleviating the disease (Chen et al., 2016; Yan et al., 2020). At day 20, the increasing trend of IFN-γ and IL-17 discontinued. It is suggested that the experimental group can clear the pathogen in the early stage, while the control group can not induce enough immune response in the early stage. Therefore, the inflammation will not heal and aggravate the tissue damage in the control group.

Although the antifungal mechanism of AMPs remains unclear, a widely accepted theory is the cell membrane damaging effect, positively charged AMPs and negatively charged microbial cell membranes show electrostatic attraction. AMPs cause leakage of intracellular water-soluble substances by destroying the fungal plasma membrane, eventually leading to lysis and death of the fungi (Delattin et al., 2017; Polesello et al., 2017). In addition, AMPs can work on fungi in many ways, including membrane action and interaction with important organelles, such as mitochondria and nucleic acid macromolecules in fungal cells, eventually causing fungi death (Delattin et al., 2017; Polesello et al., 2017). Due to their low drug resistance and low toxicity, AMPs show corresponding research value and development potential. Microorganisms have a very low probability of developing resistance to AMPs. This is because AMPs bind to the microbial cell membrane by electrostatic attraction, meaning that microorganisms must change their membrane structure to avoid AMP attacks (Lazzaro et al., 2020). However, the negative charge of the microbial cell membrane is the result of evolution over hundreds of millions of years; it is an important conservative membrane component widely found in microbial communities. Therefore, it is particularly difficult for microorganisms to become resistant to AMPs.

Phage particles can remain stable for a long period and are highly resistant to physical and chemical factors (Sharma et al., 2017). This method is highly applicable in both experimental studies and clinical applications. Additionally, phage vectors show good immunogenicity. Small synthetic peptides usually bind to large vectors or require adjuvants to effectively trigger immune responses. However, phage vectors can produce intense immunoreactions without adjuvants. In addition, we propose a new method for the preparation of AMPs, phage displays. In our experiment, we used the self-synthesis assembly and release of phages to produce many AMP-displaying recombinant phages. Through the binding of recombinant phage with phage antibodies (Figure 1), AMP antibodies react with recombinant phage PG, indicating that the specific reaction of the antibodies with recombinant phage is with AMPs in recombinant phage rather than phage components.

ITR-resistant *S. globosa* strains have also been reported (Vettorato et al., 2018). The use of PG as an adjuvant treatment can lead to an overall improvement in the efficacy of antifungal chemotherapy, as well as a reduced duration of antifungal treatment and reduced inflammation reaction. Moreover, with a reduced duration of antifungal drug application, PG may avoid the adverse side effects of the drugs, especially in patients with liver dysfunction, pregnant women, and children who are not suitable for antifungal drug therapy (Kauffman et al., 2007).

## Conclusion

The results of this study demonstrated that the recombinant phage effectively inhibited the growth of *Sporothrix globosa* by activating fungal apoptosis. During this process, the morphological structure, MMP, ROS accumulation, and metacaspase activation of *Sporothrix globosa* cells changed significantly. Moreover, PG induced the expression of IFN-γ and IL-17, and its therapeutic effect on mice infected with *Sporothrix globosa* was almost equivalent to that of ITR. In conclusion, our results suggest that the application of phage-based nanomaterials displaying ToAP2D peptides may be a new, safe, and effective method for the treatment of *Sporothrix globosa* infection.

## Data Availability

The original contributions presented in the study are included in the article/supplementary materials, further inquiries can be directed to the corresponding author.
