# Supplementary material for: Recombinant phage displaying ToAP2D peptide with antifungal activity against Sporothrix globosa
Source: Front Pharmacol. 2022 Oct 7;13:1022651. doi: 10.3389/fphar.2022.1022651 (PMC9585229; doi:10.3389/fphar.2022.1022651)
Supplement: Supplementary file 1 [file DataSheet1.PDF]

Recombinant Phage Displaying ToAP2D Peptide with Antifungal Activity against Sporothrix globosa

Running title: Activity of a new ToAP2D peptide

Tianyi Yan[1], Lin An[2], Feng Chen[2,*]

[1]China-Japan Union Hospital of Jilin University, 126 Xiantai Street, Changchun 130033, Jilin Province, People's Republic of China

[2]Department of Dermatology, China-Japan Union Hospital of Jilin University, 126 Xiantai Street, Changchun 130033, Jilin Province, People's Republic of China

***Corresponding author:** Feng Chen

Department of Dermatology, China-Japan Union Hospital of Jilin University, 126 Xiantai Street, Changchun 130033, Jilin Province, People's Republic of China

Email: chenfengdoctor@jlu.edu.cn;

Tel:+8613504431325.

Supplementary Figures and Legends

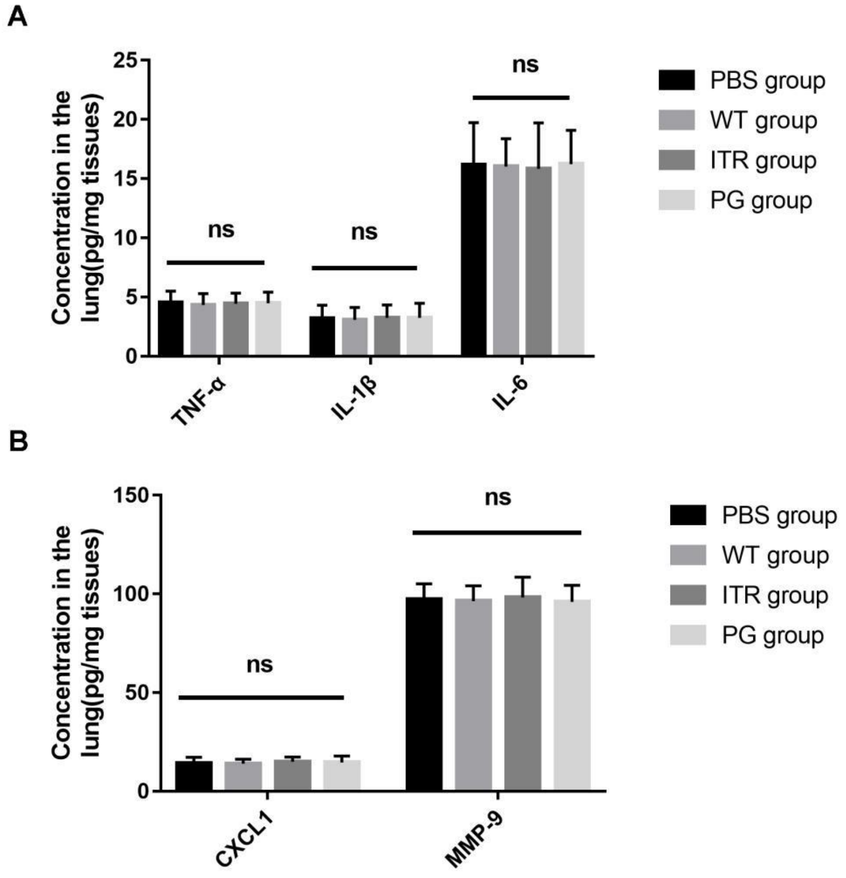

sFig1. After PG, WT or ITR challenge, the levels of TNF-a, IL-1β, IL-6, CXCL1, and MMP-9 production in the bronchoalveolar lavage fluid (BALF) was compared with the PBS group. (A) TNF-a, IL-1β and IL-6 levels in the BALF was measured by ELISA. (B) CXCL1, and MMP-9 in the BALF was measured by ELISA. Compared to the levels in mice from the PBS group, ns: no significance.WT: wild-type phage injection; ITR: itraconazole injection; PG: phage-GK injection.

.

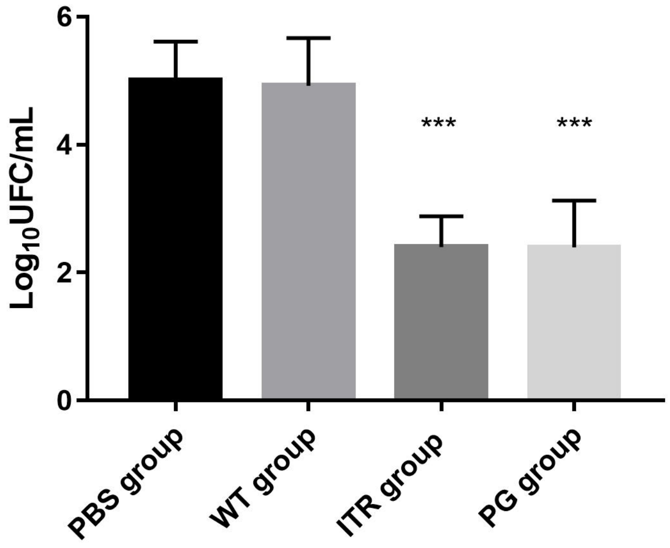

sFig 2. Log_{10} CFU in mouse lung. To determine whether PG, WT or ITR challenge could reduce the S.globosa levels in organs, the mice was sacrificed, and the Log_{10} CFU was quantified in lung on day 20.There were significantly fewer Log_{10} CFU in the mice at PG and ITR group than the mice in the PBS group. However, no significant difference was observed between the WT and PBS group. Compared to the levels in mice from the PBS group, ***$p < 0.001$.WT: wild-type phage injection; ITR: itraconazole injection; PG: phage-GK injection.
